# Evaluating quality of life in pediatric palliative care: a cross-sectional analysis of children’s and parents’ perspectives

**DOI:** 10.1007/s00431-023-05330-4

**Published:** 2023-12-19

**Authors:** Daniel Toro-Pérez, Joaquin T. Limonero, Montserrat Guillen, Catalina Bolancé, Sergi Navarro Vilarrubí, Ester Camprodon-Rosanas

**Affiliations:** 1 School of Psychology, Stress and Health Research Group, Autonomous University of Barcelona, Barcelona, Spain; 2 Palliative Care and Complex Chronic Patient Service (C2P2), Sant Joan de Déu Hospital in Barcelona, Barcelona, Spain; 3 Children and Adolescent Mental Health Research Group, Child and Adolescent Psychiatry and Psychology Department, Hospital Sant Joan de Déu, Barcelona, Spain; 4 ANJANA Working Group, Catalan-Balearic Society of Palliative Care, Catalan Society of Pediatrics, Barcelona, Spain; 5 Department of Econometrics, Statistics and Applied Economics, University of Barcelona, Barcelona, Spain

**Keywords:** Quality of life, Pediatric palliative, Children, Parents, Life-limiting conditions, Psychological assessment

## Abstract

The patient’s perspective is an essential component of understanding the individual experience of suffering in children with palliative needs, but it is a perspective that is often overlooked. The aim of this study was to compare the perception of quality of life (QoL) of children with life-limiting and life-threatening conditions expressed by the children themselves and their parents. Through a cross-sectional study, the responses of 44 parent–child dyads were obtained and the analysis was performed with the statistics based on Student’s *t* distribution and non-parametric tests. Children value QoL more positively (mean = 6.95, SD = 1.85) than their parents (mean = 5.39, SD = 2.43). This difference exists even if we consider sociodemographic and disease variables. The presence of exacerbated symptoms is the situation in which both parents (mean = 3.70; SD = 1.95) and children (mean = 5.60; SD = 1.17) evaluate QoL more negatively.

*Conclusions*: Children have a more optimistic view than their parents. When the child is the one who reports a lower QoL score than their parent, the child should be carefully monitored. The voice of the child and that of the family members can be collected to create a “family voice” and can be complementary.

**Table:** 

**What is Known:** *• Children with life-limiting conditions experience multiple and changing symptoms that affect their QoL.* *• The child’s perspective is often overlooked.*	
**What is New:** *• Children value QoL more positively than their parents do, even if we control for sociodemographic variables and the disease itself.* *• When the child is the one who reports a lower QoL score than their parent, the child should be carefully monitored.*

**What is Known:**

*• Children with life-limiting conditions experience multiple and changing symptoms that affect their QoL.*

*• The child’s perspective is often overlooked.*

**What is New:**

*• Children value QoL more positively than their parents do, even if we control for sociodemographic variables and the disease itself.*

*• When the child is the one who reports a lower QoL score than their parent, the child should be carefully monitored.*

## Introduction

Worldwide, there are approximately 21 million children and young people aged 0–19 years (hereafter “children”) with chronic complex conditions and life-limiting and life-threatening conditions (hereafter “LLTC”) [1]. The World Health Organization defines pediatric palliative care (PPC) as a holistic approach focusing on improving the quality of life of children with LLTC and their families [2, 3], improving symptoms and concerns, easing suffering, and supporting families in delineating their goals of care and making decisions accordingly [4–8].

Most of the children with LLTC have several significant chronic health problems that affect multiple organ systems and result in functional limitations, and the treatment of these children is complex and extends over a long period of time [9], and they face a multitude of challenges [10], which can negatively impact their QoL [11, 12]. As a result, multidisciplinary assessment (physical, psychological, social, and spiritual) [13, 14] is critical and necessary for comprehensive and accurate evaluation of a patient’s suffering and for decision-making [15, 16].

Inquiring into QoL fosters insight into the effect of disease trajectory on a child and overall perception of lived experience [17]. Although including the child’s perspective is an essential component of understanding the individual experience of suffering, it is a perspective that is often overlooked [18, 19]. Childhood and adolescence are life stages with many developmental changes, and yet, it remains unclear how these changes influence QoL in young people, particularly for those with chronic illness [20]. Measuring care, outcomes, and experiences during end of life is challenging but patient-reported outcome measures (PROMs) can be used [21]. The research team confirms that, in practice, the evaluation of the QoL is usually interpreted by the professionals through the QoL indicated by the parents.

The aim of this study was to compare the perception of quality of life of children with palliative needs expressed by children themselves with the perception of the child’s quality of life that is expressed by their parents. Our hypothesis is that there will be differences between the perception of children and that of their parents.

## Methods

### Study design and setting

This is a cross-sectional study of the perceived quality of life of the child by the children as stated by themselves and by their parents. In this study, we used a convenience sample of children being treated at the Palliative Care and Complex Chronic Patient Service (C2P2) of the Sant Joan de Déu Hospital (Barcelona, Spain). We evaluated the QoL of all the children through assessments, attempting to gauge the opinions of the children and their parents from June 2021 to February 2023. The study was approved by the Medical Research Ethics Committee of the Sant Joan de Déu Hospital (reference code PIC-158–20 in 02/06/2020).

### Study sample

Children attended by C2P2 and their parents were eligible to participate. Additional inclusion criteria were as follows: (1) having a minimum age of 9 years and fluency in Spanish or Catalan language, (2) the child’s first multidisciplinary evaluation had been carried out by C2P2’s team, (3) the child’s psychologist referent should validate the participation on the study, (4) the father and/or mother were the child’s legal tutors, and (5) the parents and the child had signed the consent form. Children with moderate or severe neurological impairment or situation of imminent death were excluded.

### Variables and data collection

Senior psychologists from the palliative care service collected data via interviews with the participating children and their parents. Sociodemographic and disease variables were recorded by medical history and health professional questions; child and parents report about their perception of children’s QoL. After giving their written informed consent, the child and their parents evaluated the child’s QoL*.* Parents provided their QoL evaluation and health professionals answered specific questions about disease variables within a maximum 3-day period to ensure that the time elapsed since the child’s response did not result in a confounding variable, thus avoiding potential sources of bias.

Sociodemographics information: Age, gender, school attendance, family structure, and presence of sibling were collected from the medical history.

Disease variables: Data collected included diagnosis, adequacy of the therapeutic effort (the decision to withhold or withdraw diagnostic and therapeutic measures in response to the patient’s condition, avoiding potentially inappropriate behaviors and redirecting treatment goals towards comfort and well-being), presence of exacerbated symptoms and time since diagnosis from medical history, and specific questions answered by health professionals.

Child’s perceived quality of life*:* An adaptation to distress thermometer (DT) was used [22, 23] which is a one-item instrument indicating a patient´s general distress level on a 0–10 visual analogue scale. Children and parents were asked: “In general, how do you rate your quality of life (well-being) at the present time?” The response ranged from 0 to 10, where 0 corresponds to very bad and 10 to very good. There is only one evaluation from parents on their child’s QoL.

### Statistical analysis

The database is obtained from three sources, the children’s medical history, the perception of children’s QoL obtained from the parent’s responses, and the children’s response to the QoL question. The 0–10 scale of evaluation of QoL has been treated as interval scale. The database was created and analyzed using the software R version 4.2.1. Basic descriptive statistics are obtained, the mean and the standard deviation for quantitative variables and the frequency distribution for categorical variables. Differences between groups for independent and paired data are tested by comparing the means of the groups with the statistics based on Student’s *t* distribution (*t*-test). We based on the Jarque–Bera test for the skewness and kurtosis of the normal distribution for children’s and parents’ groups. Additionally, non-parametric tests for comparing independent groups (Mann–Whitney–Wilcoxon test) and for paired data (Wilcoxon signed-rank test) are implemented. *p*-values are reported, and conclusions are drawn with a 95% confidence level. The Bonferroni correction was implemented for multiple analyses.

## Results

**Fig. 1:**
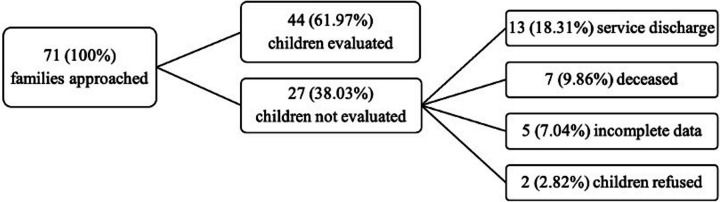
Flow chart of children evaluated

Figure 1 is a flow chart representing the participants in the study. Among the 71 families who were approached to participate in the study, 13 (18.31%) were excluded because patients were discharged before the study could be carried out, 7 (9.86%) patients were deceased, 5 (7.04%) could not be fully assessed by all parties within the maximum 3-day evaluation window, and 2 families (2.82%) refused to participate. Finally, 44 child-parent dyads were analyzed in the final sample.

Table 1 presents the description of the final sample of 44 pediatric patients. The mean age of children is 15.6 years (standard deviation (SD) = 4.2; range: 0 < age < 21), 14 females (31.8%), and 30 males (68.2%). Twenty-five children (56.8%) attend school; most of them have a family structure of coexistence with 2 parents (65.9%) and presence of siblings (59.1%). Regarding the variables related to the disease, oncohematological diseases are present in 65.9% of the cases and we also find dermatopathies like epidermolysis bullosa (18.2%), respiratory system diseases (6.8%), and other diseases in 9.1%; on average, children have been diagnosed 6.4 years (SD = 5.1) previous to the study. Regarding the disease situation, 65.9% are patients with an adequacy of therapeutic effort. Symptoms are stable for 34 children (77.3%) and exacerbated for 10 children (22.7%). In our sample data, 11 (25%) evaluations were provided by the father and 33 (75%) were provided by the mother.

**Table 1:** Sociodemographic and disease characteristics of the patients (N = 44)

**Sociodemographic data**	
Characteristic	*n* (%)
** Age**	
Mean (SD)	15.60 (4.17)
Median	14.5
** Gender**	
Male	30 (68.18)
Female	14 (31.82)
** Gender of parents**	
Male	11 (25.0)
Female	33 (75.0)
** School attendance**	
Yes	25 (56.82)
No	19 (43.18)
** Biparental family**	
Yes	29 (65.91)
No	15 (34.10)
** Siblings**	
Yes	26 (59.09)
No	18 (40.91)
** Disease data**	
Characteristic	*n* (%)
** Oncohematology**	
Yes	29 (65.91)
No	15 (34.09)
** Adequacy of therapeutic effort**	
Yes	29 (65.91)
No	15 (34.09)
** Exacerbated symptoms**	
No	10 (22.73)
Yes	34 (77.27)
** Years diagnosed**	
Mean (SD)	6.41 (5.09)

### Difference between children and their parents

Children rate their quality of life with a mean of 6.95 (SD = 1.85, median = 7.00) and their corresponding matched parents with a mean of 5.39 (SD = 2.43, median = 6.00). Means and standard deviations are shown in Table 2. The results regarding the comparison of the scores between the children and the parents when they rate children’s QoL (Table 2) reveal that there is statistically significant difference in the mean scores by “years diagnosed” (*p* = 0.037) in the children’s group and when exacerbated symptoms are present both in the children’s group (*p* = 0.001) and in the parents’ group (*p* = 0.008).

**Table 2:** Quality of life evaluation 0 (bad)–10 (very good) of patients (N = 44) by informant

			Child		Parents	
		*n*	Mean (SD, median)	*p*^(*)^	Mean (SD, median)	*p*^(*)^
**Sociodemographic data**			(a)		(b)	
All patients		44	6.95 (1.85, 7.00)	–	5.39 (2.42, 6.00)	–
Age	≤ 14 years	22	7.41 (2.06, 7.50)	0.105	5.50 (2.50, 6.00)	0.760
	> 14 years	22	6.50 (1.54, 6.50)		5.27 (2.39, 5.50)	
Gender	Male	30	7.23 (1.94, 7.00)	0.118	5.57 (2.30, 6.00)	0.506
	Female	14	6.36 (1.55, 7.00)		5.00 (2.72, 5.50)	
Gender of parents	Male	11	–	–	4.82 (2.40, 5.00)	0.825
	Female	33	–		5.56 (2.44, 6.00)	
School attendance	Yes	25	7.08 (1.80, 7.00)	0.617	4.88 (2.22, 5.00)	0.121
	No	19	6.79 (1.96, 7.00)		6.05 (2.57, 6.00)	
Biparental family	Yes	29	7.17 (1.71, 7.00)	0.319	4.97 (2.53, 5.00)	0.089
	No	15	6.53 (2.10, 7.00)		6.20 (2.04, 6.00)	
Siblings	Yes	26	6.77 (1.77, 7.00)	0.443	5.54 (2.32, 6.00)	0.631
	No	18	7.22 (1.99, 7.50)		5.17 (2.62, 5.50)	
**Disease data**						
Oncohematology	Yes	29	6.55 (1.72, 7.00)	0.055	5.62 (2.31, 6.00)	0.404
	No	15	7.73 (1.91, 8.00)		4.93 (2.66, 5.00)	
Adequacy of therapeutic effort	Yes	29	6.66 (1.65, 7.00)	0.177	5.55 (2.25, 6.00)	0.566
	No	15	7.53 (2.13, 8.00)		5.07 (2.79, 5.00)	
Exacerbated symptoms	Yes	10	5.60 (1.17, 5.50)	0.001	3.70 (1.95, 4.00)	0.008
	No	34	7.35 (1.84, 7.50)		5.88 (2.35, 6.00)	
Years diagnosed	≤ 5 years	24	6.42 (1.64, 6.50)	0.037	5.29 (2.31, 6.00)	0.783
	> 5 years	20	7.60 (1.93, 7.50)		5.50 (2.61, 6.00)	

^(*)^*p*-value. Two-sided *t*-test of mean difference between two groups of patients

The one-sided *t*-test for the difference in means for paired samples reveals a mean assessment of children higher than that of parents (difference = 1.57, *t*-test = 4.31, *p* < 0.001). We cannot reject normality (*p* = 0.689 and *p* = 0.863 for children and parents, respectively). Table 3 shows that the average difference made in the evaluation of the QoL between a child and their parents is maintained after considering the different sociodemographic and clinical variables. Higher average scores of children compared to parents are found independently of the age, gender, school attendance, siblings, diagnosis, adequacy of therapeutic effort, exacerbated symptoms, and years diagnosed. Regarding the gender of the parent who evaluates, no statistically significant differences have been found either (*p* = 0.825). No difference is found in the mean score of QoL provided by children with no biparental family (*p* = 0.270).

**Table 3:** Difference between the score given by children minus the score given by their parents

		Difference			Wilcoxon signed-rank	
	*n*	Mean	*p*^(*)^	*t*-test	*p*^(*)(**)^	*W*
**Sociodemographic data**						
All patients	44	1.57	< 0.001	4.307	< 0.001	571.5
Age						
≤ 14 years	22	1.91	0.004	2.963	0.005	158.5
> 14 years	22	1.23	< 0.001	3.594	0.002	137.5
Gender						
Male	30	1.67	< 0.001	3.505	0.001	275.5
Female	14	1.36	0.013	2.510	0.009	59.0
School attendance						
Yes	25	2.20	< 0.001	3.930	< 0.001	225.0
No	19	0.74	0.022	2.163	0.024	83.5
Biparental family						
Yes	29	2.21	< 0.001	5.023	< 0.001	291.0
No	15	0.33	0.270	0.627	0.331	44.5
Siblings						
Yes	26	1.23	0.002	3.226	0.002	180.5
No	18	2.06	0.005	2.946	0.007	115.5
**Clinics**						
Oncohematology						
Yes	29	0.93	0.005	2.799	0.007	217.5
No	15	2.80	0.001	3.609	0.002	86.0
Adequacy of therapeutic effort						
Yes	29	1.10	< 0.001	3.500	0.002	216.5
No	15	2.47	0.006	2.902	0.008	91.0
Exacerbated symptoms						
Yes	10	1.90	0.006	3.143	0.007	43.0
No	34	1.47	0.001	3.348	0.001	311.5
Years diagnosed						
≤ 5 years	24	1.13	0.018	2.229	0.019	146.0
> 5 years	20	2.10	< 0.001	4.098	0.000	148.5

^(*)^*p*-value: paired sample one-sided test of mean difference between the patient’s and the parents’ evaluation

^(**)^If data have ties, *p*-values are approximated

### Specific differences by child-parent dyads

**Fig. 2:**
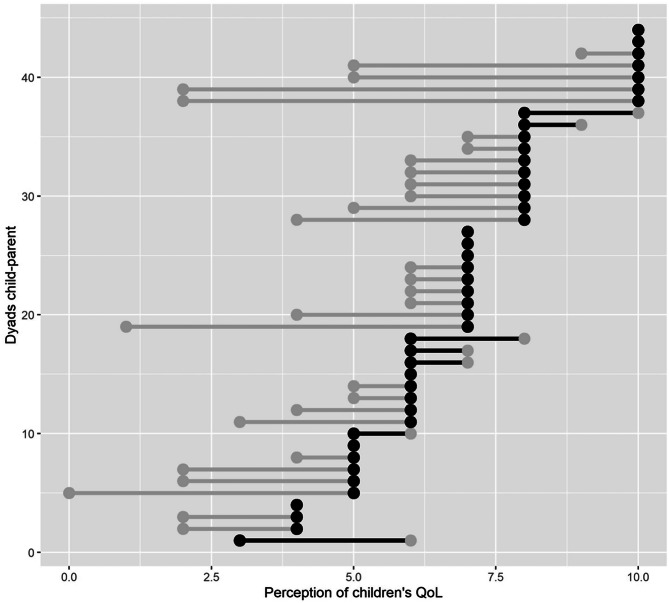
Perception of children’s QoL made by each parent–child dyad

Figure 2 represents the perception of children’s quality of life made by each parent–child dyad. The vertical axis represents the 44 child-parent dyads, and the horizontal axis represents the score that each one has made, with the black dots being the children’s score and the white dots that of the parents. We found that in 7 dyads (15.9%), the children evaluate their QoL more negatively than the parents.

## Discussion

In line with the main objective of the study, significant differences were found on the assessment of children with LLTC’s QoL made by children and that of their parents. Children with LLTC value QoL more positively than their parents do, even if we control for sociodemographic variables and the disease itself. The QoL responses were treated as interval data, but the QoL can be considered an 11-point Likert scale and median differences be tested accordingly.

### Parents’ worst perception, optimistic children

These results replicate previous findings of differences in parent–child agreement of reported QoL [24–26]. Parents report a higher prevalence of physical and psychological symptoms than children. The overestimation of symptom prevalence was most significant for the physical symptoms of fatigue, nausea, and lack of appetite and for the psychological symptoms of feeling nervous and sadness [27, 28]. Other studies confirm that the majority of the symptoms that parents tended to overestimate were psychosocial concerns [27] possibly influenced by the suffering that the parents are enduring.

Children may embrace optimism as a coping strategy and so find it difficult to negatively evaluate their well-being and QoL [29]. Denial, repression, and hope can be used adaptively by children with chronic illnesses to maintain a compromise between the ego ideal and the realistic self-concept [30]. The expression of a good quality of life and the maintenance of normality can reduce the feeling of helplessness and despair [31]. With our results, we observe that the interests of the child are, ultimately, different from the interests of adults, even when we measure them with instruments similar to those of adults. Our results confirm something already known in pediatrics: “children are not just small adults.”

### Sociodemographic variables

In our study, we found that sociodemographic variables such as the origin of the child or the type of household have some kind of influence on the children’s QoL expressed by children and parents. More studies are needed in this regard, but it is coherent that sociocultural factors, including beliefs and religion, could have an influence on the concordance on between perceptions of children and parents [27]. No significant differences were found according to the gender of the parents. It is an interesting topic of study in which some studies [32] have found that parents were more likely to both mention and match each other on some problem and hope topics such as physical health and the child’s quality of life.

### Disease’s variables

On the other hand, static labels such as the diagnosis of the disease or the adequacy of the therapeutic effort have no direct relationship with the perception of children’s QoL neither by children nor by parents. Other studies do find a direct relationship with the diagnosis of the disease [18, 33], something that the researchers assumed was difficult to observe in our study due to the large number of diagnoses and the size of the sample. Although it is not a variable that has been significant in our model, some studies show the importance of the time since diagnosis, which can influence the perception of QoL in both directions: decreasing it because they have memories of a better previous life that they want to recover or increasing it, since a longer duration of the disease gives more time for psychological adaptation, but may also lead to an accumulation of negative illness-related consequences [34–36].

### The importance of the exacerbated symptoms: perception like healthy or ill child

The presence of exacerbated symptoms is shown to be a significant and influential variable in the perception of a worse QoL for both children and parents. Other studies find that disease progression is associated with higher physical and psychological scores [18, 33, 37]. Exacerbated symptomatology is possibly related to the effects of the treatments and moments of relapse [33]. We understand that the presence of intensified symptoms can directly affect children with LLTC not only in the physical experience of suffering but also in loss of autonomy and increased dependency, thus reducing child’s QoL and emotional well-being. However, one primary explanation for interpreting these results is that children perceived themselves as healthy, except during episodes of illness [37]. In children with LLTC, there may be an effect of adaptation to the disease situation that is altered when disruptive situations occur, such as aggravated symptoms already described as a source of suffering [38], which confront children with their reality of “being sick” and directly affect their QoL and emotional well-being.

### Possible implications in clinical practice

Detecting extreme cases in these discrepancies has a clinical implication. When the difference is negative, i.e., whenever children value their QoL more negatively than their parents, this could be a signal indicating that the child is experiencing suffering and requires further attention. In addition, parents may project emotions based on their own expectations, so that high discrepancies when the child’s are high and their parents are low tell us about the suffering of parents and their need for psychological care. We cannot ignore the fact that in pediatric palliative care, parents are exposed to emotionally demanding clinical experiences with high levels of bonding and involvement when the suffering of a child or a family member is particularly intense [39, 40]. In fact, the suffering of the patient affects the suffering of their relatives and vice versa. This interaction is known as reciprocal suffering [41].

Regardless of the circumstances, clinicians need to be aware of the potential for discordance in children’s and parents’ assessments of children’s symptom experiences [27]. It is most important to let children with palliative needs say what they think, answer for themselves, and express their feelings, as well as assess their own QoL [35], and that we use validated tools with good psychometric properties that can be used in daily clinical practice. The ability to understand the experience of symptoms improves when professionals integrate the child’s perspective through self-reported outcomes [28]. Hinds et al. [42] noted that the ‘‘child’s voice’’ should not replace information from parents and clinicians. The child’s voice and those of family members can be compiled to create a ‘‘family voice” and can be complementary. The authors consider that these are preliminary reflections that should be investigated in depth.

## Strengths and limitations

Despite the importance of the findings reported here, this study has limitations that merit consideration. One limitation is related to the sample size. Studies are needed that include more children and parents, albeit acknowledging the inherent difficulty of palliative situation in pediatric patients. Even so, carrying out a prospective study with a sample of 44 child-parent dyads of children with LLTC is also considered by the authors as a strength in the population being treated.

Another limitation that we may encounter is the arbitrary establishment of decisions such as the type of differentiation according to age or the adequacy of the therapeutic effort. The research team considers that the impact that these variables have and their implication on the perception of quality of life must be delved deeper into. It is also necessary to delve deeper into the impact of parental sociodemographic variables, such as gender, and look at the influence of having both parents evaluate whenever possible.

The authors are aware that simplifying the QoL to a single question is risky. We know that health-related QoL measurement instruments must consist of physical, social, and mental health dimensions as outlined by the World Health Organization. The tool used provides a simple evaluation of the QoL and allows having a concrete and easily reproducible measure to be used continuously that is useful to healthcare professionals in daily practice. This also leads us to consider the importance of using this type of scale as a comparative measure for the same subject at different times and not to compare between subjects. That is why we need tools to measure the QoL of children in a more continuous way, useful for clinical practice, and that is far from complex. Likewise, our study had a cross-sectional design. To confirm these results, it would be appropriated to carry out longitudinal studies with the participation of a greater number of health centers to avoid evaluative biases.

## Conclusion

Despite the difficulties inherent in advanced and end-of-life disease in the pediatric population, children with LLTC can assess their perception of quality of life. Compared to the assessment made by children and their parents, parents provide a significantly lower score. That difference exists even if we consider sociodemographic and disease variables. The presence of exacerbated symptoms is the situation in which both informants, parents and children, evaluate the quality of life more negatively. The dyad of informants (children and parents) must be seen and treated as complementary in order to establish the best possible evaluation of the children’s quality of life. When the child is the one who reports their QoL score lower than the parents QoL score of the child. A lower score on children’s side may be a sign of distress that requires further analysis in case additional support is needed.

## Data Availability

Toro-Pérez, Daniel; Limonero, Joaquin T.; Guillen, Montserrat; Bolance, Catalina; Navarro-Vilarrubí, Sergi; Camprodon-Rosanas, Ester (2023), “QoL pediatric palliative care dataset”, Mendeley Data, V1, 10.17632/m479gs98vz.1.

## Abbreviations

Children: Children and young people
LLTC: Life-limiting and life-threatening condition
Parents: Parents and careers
